# Biobanking and use of gonadal tissues - a promising strategy for conserving wildlife from the Caatinga biome

**DOI:** 10.1590/1984-3143-AR2022-0135

**Published:** 2023-02-13

**Authors:** Alexandre Rodrigues Silva, Alexsandra Fernandes Pereira, Pierre Comizzoli

**Affiliations:** 1 Laboratório de Conservação de Germoplasma Animal, Universidade Federal Rural do Semi-Árido, Mossoró, RN, Brasil; 2 Laboratório de Biotecnologia Animal, Universidade Federal Rural do Semi-Árido, Mossoró, RN, Brasil; 3 Smithsonian National Zoo and Conservation Biology Institute, Washington, USA

**Keywords:** biodiversity, wildlife conservation, genome resource banking, ovarian tissue, testicular tissue

## Abstract

Biological Resource Banks (BRB) or Genetic Resource Banks (GRB) are critical tools for the conservation of animal biodiversity. According to the International Union for Conservation of Nature, more than 38,500 species are threatened with extinction, out of a total of 138,300 surveyed species. These banks are repositories of biological samples and data recovered and preserved for the long term by zoos, universities, research centers and other conservation organizations. In recent years, BRB have increasingly included ovarian and testicular tissues as additional options to rescue and propagate wild species, especially those at risk of extinction. After in vitro culture or grafting, gonadal tissues are potential sources of matured gametes that can be used for Assisted Reproduction Technologies while informing about gametogenesis or mechanisms involved in infertility. It therefore is crucial to properly recover, cryopreserve, and culture these tissues using species-specific protocols. Developing BRBs is currently one of the strategies to preserve species from the Caatinga biome - an exclusively Brazilian biome with a rich wild fauna that suffers from anthropogenic activities. Among wild species from this biome, studies have been primarily conducted in collared peccaries, agoutis, cavies, and armadillos to preserve their ovarian and testicular tissues. Additionally, domestic species such as the domestic cat and donkeys have been proposed as models for wild species that are phylogenetically close. This review addresses the main technical aspects involved in obtaining BRB derived from gonadal tissues in some wild species of the Caatinga biome. It reports recent advances and perspectives to use these biological materials for wildlife conservation.

## Introduction

Although the extinction of species is an irreversible process and is the result of natural evolution, anthropogenic activities have considerably accelerated extinctions of many species (Singina et al., 2014). According to data from the Red List of the International Union for Conservation of Nature, of a total of 138,300 species surveyed, more than 38,500 species are threatened with extinction, 26% of which are mammals (IUCN, 2022). In the current situation, zoos, universities, research centers and other conservation institutions have become important organizations for the development of conservation strategies for wildlife species (Pizzutto et al., 2021, Stadtländer, 2022). Among such important conservation strategies, the role of Biological Resource Banks (BRB) in the establishment of genetic resources and the conservation of endangered species is particularly evident in the last decade (Singina et al., 2014; Holt and Comizzoli, 2021).

The BRB or Genetic Resource Banks are considered sources of gametes, embryos, gonadal tissues, and somatic cells after recovery, processing, and cryopreservation for long term storage (León-Quinto et al., 2009). Though most banks store semen (Moreira et al., 2022) and embryos (Souza-Fabjan et al., 2022), the interest in BRB composed of gonadal tissues has grown over the years, especially with the rapid loss of many wild species (Campos et al., 2019a, Fernandes et al., 2023). In general, these banks can be used in conservation efforts to produce fully-grown gametes, aiming at their use in Assisted Reproduction Technologies (ARTs), such as *in vivo* and *in vitro* embryo production, as well as providing reproductive cells for *in vitro* toxicity testing. Moreover, studies related to gonadal tissues allow to understand the developmental biology of gametes, as well as understanding mechanisms involved in infertility.

Protocols to create BRB from ovarian and testicular tissues have been developed in different wild species (Campos et al., 2019b, Praxedes et al., 2020, Silva et al., 2021a ,b, 2022). In general, the efficiency of BRB of preantral follicles (PAFs) enclosed in ovarian tissues or germ cells enclosed in testicular tissues depend on the technical conditions involved in the recovery, cryopreservation, *in vivo* or *in vitro* culture and characterization of these tissues, which are all species-specific (Campos et al., 2019b, Silva et al., 2020). BRBs have been developed in species from the Caatinga biome, which is characterized as an exclusively Brazilian biome with a rich wild fauna that suffers from anthropogenic activities (Silva et al., 2010). In the Caatinga biome, a total of 148 species of mammals have already been cataloged, such as collared peccaries (*Pecari tajacu*), red-rumped agoutis (*Dasyprocta leporina*), Spix’s yellow-tooth cavies (*Galea spixii*), and six-banded armadillos (*Euphractus sexcinctus*). Preservation of ovarian and testicular tissues have already been attempted in those species. Additionally, domestic species such as the donkeys (Lopes et al., 2018) and domestic cat (Amelkina et al., 2022) have been proposed as models for wild species that are phylogenetically close.

The objective of this review is to address the main technical aspects involved in obtaining BRB derived from gonadal tissues in some wild species of the Caatinga biome. It also reports advances and perspectives to use these biological materials for wildlife conservation.

## Preservation and use of ovarian tissues

The cryopreservation associated with the *in vitro* or *in vivo* culture of PAFs represents a promising alternative for the preservation of female germplasm. Almost all (>90%) of the oocyte reserve of the ovary is found in these follicles, making it possible to preserve genetic material from a single female, (i) before it goes to atresia, (ii) from individuals at any stage of development (fetus, pre-pubescent, and adult) and (iii) individual status (alive animals or post-mortem) (Silva et al., 2015). This technology allows the creation of BRB with biomaterials form females of high genetic value that suddenly die, or females that, for medical reasons, have to undergo ovariectomy (Campos et al., 2019a). In addition to allowing the storage of a homogeneous population of oocytes from the same individual, it allows the study of factors related to follicular development, contributing to the elucidation of the role of toxic substances, for instance.

Given their importance and prospects for use, several protocols for cryopreservation and culture of PAFs have been developed in different species, with promising results. In general, studies on PAFs enclosed in ovarian tissues involve studying the ovarian features, conditions for transportation, cryopreservation, *in vitro* and *in vivo* culture of tissues. In collared peccaries, red-rumped agoutis, Spix’s yellow-tooth cavies, armadillos, and Northeastern donkeys, significant advances have been achieved (Table 1).

**Table 1:** Advances and main references in ovarian tissue preservation protocols in species from the Caatinga biome.

**Species**	**Follicle population characterization**	**Cryopreservation**	***In vitro* culture**	**Ovulation post xenografting**
Six-banded armadillo	Brasil et al. (2021)	--	--	--
Northeastern donkey	Lopes et al. (2017)	Lopes et al. (2018)	--	--
Spix’s yellow-tooth cavy	Praxedes et al. (2017)	Praxedes et al. (2017)	--	--
Collared peccary	Lima et al. (2012)	Campos et al. (2019b) Lima et al. (2019)	Lima et al. (2018) Gomes et al. (2020) Campos et al. (2021)	--
Red-rumped agouti	Santos et al. (2018)	Wanderley et al. (2012) Praxedes et al. (2020) Praxedes et al. (2021)	Praxedes et al. (2021)	Praxedes et al. (2018b)

### Studying the ovarian features

The first step in the elaboration of a preservation protocol for PAFs included in ovarian tissues consists in understanding the characteristics of these tissues in the species of interest. Studies in different species have reported differences in the population of PAFs as well as in the percentages of follicular categories (Table 2). These characteristics probably play a key role in the success of the cryopreservation and culture protocols. Furthermore, the quantity and quality parameters of the PAF population define reproductive potential (Doležel et al., 2004). In collared peccaries (Lima et al., 2012), the PAF population found (33 273 PAFs per ovary) is very low compared to the domestic swine (the domestic species most closely related to them), in which an ovarian population of 420 000 primordial follicles per pair of ovaries was reported (Gosden and Telfer, 1987). On the other hand, values found for ovarian population in collared peccaries are like those reported for goats, in which 32 204 PAFs were found per ovary (Lucci et al., 1999), indicating that these numbers could be related to the different ovulation rates among species. In addition, studies conducted under transmission electron microscopy revealed an abundance of lipids present in the ooplasm of oocytes included in peccary PAFs. Since the presence of large amounts of lipids could make cryopreservation difficult, the adoption of vitrification protocols for the preservation of PAFs in this species has been suggested (Lima et al., 2012).

**Table 2:** Characterization of preantral ovarian follicle populations in wild species from the Caatinga biome.

		**Collared peccary (** **Lima et al., 2012** **)**	**Red-rumped agouti (** **Santos et al., 2018** **)**	**Spix’s yellow-tooth cavy (** **Praxedes et al., 2017** **)**	**Six-banded armadillo (** **Brasil et al., 2021** **)**	**Northeastern donkey (** **Lopes et al., 2017** **)**
Follicle numbers	Right ovary	--	4419 ± 532	220 ± 175	--	11614 ± 3039
	Left ovary	--	5397 ± 574	195 ± 182	--	9520 ± 2523
	Total	33273 ± 579	--	416 ± 342	6175 ± 1923	21135 ± 10646
Category (in %)	Primordial	91.6	86.6	32.2	59.4	91.3
	Primary	6.3	13.0	63.7	46.3	8.2
	Secondary	2.2	0.4	4.1	2.4	0.4

Differences related to PAFs population have also been observed in species of the same infraorder. In two species of South American hystricomorph rodents, the PAF population found for red-rumped agouti was greater (Santos et al., 2018) than that reported for Spix’s yellow-tooth cavies (Praxedes et al., 2017) (Table 2); however, these cavies present a larger amount of primary than primordial follicles, different from what is commonly observed in other species.

In six-banded armadillos (Table 2), it was observed that the follicular population is significantly influenced by infection by *Mycobacterium leprae*, since the species is a natural reservoir for this microorganism that transmits leprosy (Brasil et al., 2021). In Northeastern donkeys (Table 2), it was observed that the follicular population is influenced by both age and body condition of the females (Lopes et al., 2017). Thus, these studies show that there are some particularities regarding the population and follicular category between species. This information is important in defining ovarian tissue preservation protocols.

### Short-term storage for transportation

Since many wild species are located far from specialized laboratories for cryopreservation and culture, short-term preservation of ovarian tissue during transport has been proposed, especially in collared peccaries (Lima et al., 2014). Such protocols need to maintain the viability of PAFs between the collection of biological material and its processing for cryopreservation and/or culture.

In collared peccaries, two media based on phosphate buffered saline solution (PBS) and media based on powdered coconut water (ACP®, ACP Biotechnology®, Fortaleza, Brazil) were evaluated for short-term storage of PAFs for morphology, ultrastructure, and follicular viability (Lima et al., 2014). Under refrigeration, ovarian tissues were evaluated at different times (from 6 to 36 h). In this study, ACP® provided a more adequate medium for short-term preservation of collared peccary PAFs at low temperatures compared to PBS based medium.

### Cryopreservation of ovarian tissues

The cryopreservation of ovarian tissue has been developed to increase the number of female genetic materials in BRB (Praxedes et al., 2018c). One of the main strategies for the preservation of ovarian tissue is the development of field-friendly protocols. Two methods of cryopreservation of ovarian tissue can be chosen, slow freezing and vitrification, the latter being widely applied in the field for the preservation of ovarian tissues from wild species (Table 3). Among vitrification methods, solid-surface vitrification (SSV) has been prominent for the preservation of ovarian tissues. SSV and other vitrification methods have the main advantage of reducing the formation of ice crystals and their use in the field, as they allow manipulation with low-cost materials (Carvalho et al., 2011).

**Table 3:** Studies on cryopreservation of ovarian tissues in wild species from the Caatinga biome.

**Species**	**Aim**	**Cryopreservation**			**References**
		**Technique**	**Cryoprotectants** **a**	**Main results**	
Collared peccary	(1) To establish a vitrification protocol using different cryoprotectants	Solid‐surface vitrification (SSV)	3 M EG vs. 3 M DMSO vs. 3 M DMF vs. 6 M EG vs. 6 M DMSO vs. 6 M DMF	3 M EG was more efficient for the vitrification of ovarian tissues	Lima et al. (2019)
	(2) To evaluate different vitrification methods using distinct cryoprotectants	Solid surface vitrification (SSV) method or the ovarian tissue cryosystem (OTC)	3 M EG vs. 3 M DMSO vs. 1.5 M DSMO + 1.5 M EG	Utilization of a closed system, the OTC, with 3 M EG for the vitrification of ovarian tissue	Campos et al. (2019b)
Red-rumped agouti	(1) To develop an efficient protocol for cryopreservation	Slow freezing	1.5 M EG vs. 1.5 M DMSO vs. 1.5 M PROH	1.5 M PROH was more efficient for the vitrification of ovarian tissues	Wanderley et al. (2012)
	(2) To establish a vitrification protocol using different cryoprotectants	Solid‐surface vitrification (SSV)	3 M EG vs. 3 M DMSO vs. 6 M EG vs. 6 M DMSO vs. 3 M DSMO + 3 M EG	3 M EG was more efficient for the vitrification of ovarian tissues	Praxedes et al. (2020)
	(3) To evaluate the effect of open and closed systems used for ovarian tissue vitrification on the microbiological load and preservation of follicles	Solid surface vitrification (SSV) method or the ovarian tissue cryosystem (OTC)	3 M EG	Both open and closed systems were equally efficient in preserving ovarian tissues; however, the OTC seems to provide a less adequate environment for bacterial proliferation.	Praxedes et al. (2021)
Spix’s yellow-tooth cavy	To establish vitrification using DMSO	Solid‐surface vitrification (SSV)	3 M DMSO	PAFs cryopreservation using an SSV process and 3 M DMSO	Praxedes et al. (2017)
Northeastern donkey	To compare different cryoprotectants and concentrations	Solid‐surface vitrification (SSV)	3 M EG vs. 3 M DMSO vs. 6 M EG vs. 6 M DMSO vs. 3 M DSMO + 3 M EG	3 M DMSO + 3 M EG was more efficient for the vitrification of ovarian tissues	Lopes et al. (2018)

Abbreviations: DMSO: dimethyl sulfoxide; EG: ethylene glycol; DMF: dimethyl formamide; PROH: propanediol. ^a^All cryopreservation solutions contained sucrose and fetal bovine serum.

In collared peccary (Table 3), two studies focused on the development of vitrification as a standard method for ovarian tissue preservation. Lima et al. (2019) evaluated different combinations of cryoprotectants (3 M ethylene glycol - EG vs. 3 M dimethyl sulfoxide - DMSO vs. 3 M dimethyl formamide - DMF vs. 6 M EG vs. 6 M DMSO vs. 6 M DMF). In this study, the authors observed that SSV is an effective method to preserve collared peccary female germplasm, regardless of the cryoprotectants. The use of 3 M EG appears to be the most effective cryoprotectant concentration and compound for preservation from both a viability and morphological perspective of peccary PAFs. Also, DMF appears to be a viable alternative as a cryoprotectant for mammalian ovarian tissue.

Despite being used for the preservation of the female germplasm of domestic (Carvalho et al., 2011) and wild species (Lima et al., 2019), SSV is considered as an open system that allows contact between the sample and liquid nitrogen, which represent a potential exposure to cryo-resistant pathogens. Thus, the ovarian tissue cryosystem (OTC) is an alternative closed system that has been proven to efficiently preserve ovarian tissue derived collared peccaries (Campos et al., 2019b).

For red-rumped agouti (Table 3), preservation of up to 64% PAFs was achieved using a slow freezing with different 1.5 M DMSO or EG or propanediol - PROH. However, transmission electron microscopy revealed that PROH provided the most efficient preservation of the ovarian tissue ultrastructure (Wanderley et al., 2012). Since SSV has been a promising technique in the field, it was developed in red-rumped agouti and Spix’s yellow-tooth cavy, proving to be an efficient technique in the preservation of ovarian tissues from these rodent species. For red-rumped agoutis, the cryoprotective solution composed of 3 M EG was the most efficient for the vitrification of ovarian tissues. Recently, Praxedes et al (2021) evaluated the effect of open and closed systems (SSV vs. OTC) used for ovarian tissue vitrification on the microbiological load and preservation of PAFs in the red-rumped agoutis. Authors concluded that both open and closed systems were equally efficient in preserving agouti ovarian tissues, based on PAFs morphology and DNA integrity. However, the OTC provides a less adequate environment for pathogen contamination.

For Spix’s yellow-tooth cavy (Table 3), a pioneering study on the species using SSV demonstrated that 3.0 M DMSO was efficient in preserving follicular morphology and DNA integrity (Praxedes et al., 2017). As for northeastern donkeys, the best results regarding ovarian tissue cryopreservation were achieved when using the combination of EG and DMSO in the SSV (Lopes et al., 2018).

### In vitro and in vivo culture of ovarian tissues

There are two main applications of the culture (*in vivo* and *in vitro*) of PAFs from wild species, (i) understanding the events involved in the activation, growth, and maturation of PAFs, and (ii) obtaining fully-grown gametes for use in ARTs. Several factors can influence the efficiency of *in vitro* culture, from those related to the culture system itself, to the reproductive stage of the ovary donor and categories of cultured follicles (Figueiredo et al., 2020). One key factor consists of evaluating the composition of the media, which has a direct relationship with the success of PAFs cultured *in vitro*.

In wild species from the Caatinga biome, the first studies have been carried out to identify the culture conditions involved in the development of PAFs. In collared peccaries, three studies exemplify this. Initially, Lima et al. (2018) verify the effect of follicle-stimulating hormone (FSH) supplementation to alpha minimum essential medium (α-MEM^+^) or tissue culture medium-199 (TCM199^+^) media on the *in vitro* development of PAFs by 7 days derived from collared peccaries. The FSH promotes the maintenance of the ovarian extra-cellular matrix, contributing to the follicle development. Therefore, authors suggested the use of TCM199^+^ medium supplemented of FSH for the *in vitro* development of collared peccaries PAFs under 7-day culturing conditions.

Gomes et al. (2020) confirmed that the addition of 25 ng/mL of BMP-15 to the culture medium of collared peccary PAFs maintained a high number of morphologically healthy follicles and stimulated the activation of primordial follicles after 6 days in culture. In 2021, Campos et al. observed that GDF-9, especially at a 200 ng/mL inclusion in the culture medium, was actively involved in the *in vitro* development of collared peccary PAFs. Therefore, BMP-15 and GDF-9 contribute for follicle activation and development during *in vitro* culture. These collective results improve the understanding of initial follicle development in collared peccaries and can be used to improve the protocols for *in vitro* culturing of PAFs.

For red-rumped agoutis, initial evaluations have been carried out from cultured non-cryopreserved and cryopreserved ovarian fragments. In this study, authors evaluated the cryopreservation conditions using a culture system of ovarian fragments in TCM-199 plus ITS medium (insulin 10 μg/mL, transferrin 5.5 μg/mL, and selenium 5.0 ng/mL), 0.23 mM sodium pyruvate, 2 mM glutamine, 2 mM hypoxanthine, and 1.25 mg/mL bovine serum albumin - Praxedes et al., 2021). In this species, studies aimed at identifying the most suitable components of the culture medium for PAFs are still necessary, to evaluate follicular activation and development.

In addition to *in vitro* culture, ovarian tissue grafting has been used in the evaluation of cryopreservation protocols for PAFs, allowing it to be a tool for assessing the recovery of ovarian function. In red-rumped agoutis, Praxedes et al. (2018a) evaluated the development of fresh and vitrified ovarian tissue after xenografting in female C57Bl/6 mice with severe combined immunodeficiency (SCID). The authors reported that according to the characteristics of the external genitalia and the predominance of cornified cells on vaginal cytology, ovarian activity returned in 80% of the xenografting using non-cryopreserved tissues and 17% in the xenografting using vitrified tissues. At 42 days after xenografting, follicle development and occurrence of ovulation was confirmed by the recovery and histological evaluation of the xenografted fragments. Therefore, red-rumped agouti PAFs are likely to be vitrified and subsequently resume their development through xenografting in SCID mice.

### Perspectives of preserving ovarian tissues

Among the many challenges in establishing strategies for the conservation of wild species, it is worth highlighting the fact that the reproductive physiology of many of these species remains unknown. Despite this, numerous studies have shown the efforts of researchers to fill this gap. Thus, the continuity of studies focused on the understanding of ovarian physiology and its preservation is justifiable. This perspective is evident, mainly because despite the need for improvements in the protocols for the preservation and culture of ovarian tissue, the birth of mice offspring from frozen material has already been obtained (dela Peña et al., 2002), encouraging the continuity of studies.

## Preservation and use of testicular tissues

Previously, preservation of male germplasm had been limited to well-established protocols for cryopreservation of spermatozoa obtained by ejaculation or retrieval from the epididymis. In recent years, the possibility of safeguarding testicular tissue has emerged as a promising tool for ARTs. According to this technology, the undifferentiated spermatogonia preserved into the testicular tissue could be cryopreserved and cultured, guaranteeing the unlimited production of viable spermatozoa to be used in ARTs. Therefore, the cryopreservation of testicular tissues represents an important technology for the preservation of fertility in wild mammals, enabling essential advances for the knowledge and maintenance of the species (Silva et al., 2020).

In general, protocols for germ cells enclosed in testicular tissues involves the study the testicular features, conditions for cryopreservation, *in vitro* and *in vivo* culture of tissues. Significant advances have been achieved and the establishment of protocols has allowed important results in collared peccaries, red-rumped agoutis, and Spix’s yellow-tooth cavies (Table 4).

**Table 4:** Advances and main references in testicular tissue preservation protocols in species from the Caatinga biome

**Species**	**Study of testicular features**	**Cryopreservation**	***In vitro* culture**	**Xenografting**
Spix’s yellow-tooth cavy	Santos et al. (2012), Santos et al. (2014)	Silva et al. (2019b)	--	--
Collared peccary	Costa et al. (2010)	Silva et al. (2019a), Silva et al. (2021a)	Silva et al. (2021b)	Campos-Junior et al. (2014)
Red-rumped agouti	Assis-Neto et al. (2003), Arroyo et al. (2017)	Silva et al. (2022)	--	--

### Understating the morphology and physiology of testicular tissue

Similar to what is observed in ovarian tissue, for testicular tissue technology variations in the characteristics of spermatogenesis and testicular histological parameters may result in variations in the establishment of protocols for cryopreservation and culture of testicular tissue. In general, different species have varied testicular characteristics, not only in terms of spermatogenesis, but also in terms of histological architecture and cellular characteristics. In collared peccary, for example, the daily sperm production per gram of testis in collared peccaries is approximately 23.4 ± 2 x 10^6^, which is similar to domestic and wild pigs (Costa et al., 2010). It is even necessary to point out that collared peccaries have a very peculiar testicular histological architecture, with isolated niches of Leydig cells being found in the middle of the interstitial tissue (Costa et al., 2010).

### Cryopreservation of testicular tissues

In collared peccary (Table 5), slow freezing and vitrification in cryotubes were the most suitable methods in the cryopreservation of testicular tissues derived from adult animals and using the combination of DMSO and EG as cryoprotectants in the cryopreservation solution (Silva et al., 2019a, 2021a). In these studies, glycerol-based cryoprotectants were not suitable for any of the vitrification methods employed [slow freezing, vitrification in cryotubes and SSV]. Furthermore, when other combinations of cryoprotectants [DMSO and EG alone] were evaluated, they were efficient in SSV. In these studies, all procedures were performed on adult wild animals, which are generally difficult to preserve due to their cellular diversity and vulnerability to ex vivo conditions. Although tissues from adult animals have already been cryopreserved in different species, no protocol is effective enough to obtain spermatozoa from cryopreserved testicular tissues. This fact highlights the need to continue studies to produce viable sperm and, consequently, live offspring.

**Table 5:** Cryopreservation of testicular tissues in some wild species from the Caatinga biome.

**Species**	**Aim**	**Cryopreservation**			**Authors**
		**Technique**	**Cryoprotectors^a^**	**Main results**	
Collared peccary	(1) To compare different cryoprotectants and concentrations	Solid‐surface vitrification (SSV)	3 M EG vs. 3 M DMSO vs. 1.5 EG + 1.5 M DMSO	DMSO/EG in combination is better than DMSO or EG alone for SSV of testicular tissue from peccaries	Silva et al. (2019a)
	(2) To evaluate the effects of different cryopreservation techniques including glycerol-based cryoprotectant combinations	Solid‐surface vitrification (SSV) and slow freezing (SF), conventional vitrification (CV)	1.5 M or 3.0 M [DMSO + EG vs. DMSO + Glycerol vs. EG + Glycerol]	SF and CV appeared to be the most suitable methods for the cryopreservation of adult testicular tissues DMSO + EG was more efficient for the vitrification of testicular tissues	Silva et al. (2021a)
Red-rumped agouti	To evaluate the effects of different cryopreservation techniques and cryoprotectant combinations	Solid‐surface vitrification (SSV) and slow freezing (SF), conventional vitrification (CV)	1.5 or 3 M [EG vs. DMSO vs. DSMO + EG]	The use of SSV with DMSO + EG was the best protocol for the preservation of testicular tissues	Silva et al. (2022)
Spix’s yellow-tooth cavy	To compare different cryoprotectants and concentrations	Solid‐surface vitrification (SSV)	3 M EG vs. 3 M DMSO vs. 6 M EG vs. 6 M DMSO	3 M EG was more efficient for the vitrification of testicular tissues	Silva et al. (2019b)

Abbreviations: DMSO: dimethyl sulfoxide; EG: ethylene glycol; DMF: dimethyl formamide; PROH: propanediol. ^a^All cryopreservation solutions contained sucrose and fetal bovine serum.

For this reason, steps to optimize SSV have been developed in collared peccaries and in red-rumped agoutis, with the goal of developing SSV as a safe bioprocess in the conservation of biodiversity. In red-rumped agoutis (Table 5), the use of SSV with DMSO plus EG was the best protocol for the preservation of testicular tissues from adult individuals (Silva et al., 2021b). In another hystricognath rodent - the Spix' yellow-tooth cavy (Table 5), however, the isolated use of 3 M EG f promoted the best results in the vitrification of testicular tissues (Silva et al., 2019a).

### Testicular tissue culture

The culture of testicular tissue in wild species is still a recent tool. In general, all systems have advantages and disadvantages in relation to the development of spermatogenesis, with the *in vitro* culture of testicular samples being carried out mainly in systems of organotypic culture of tissue fragments, and 2D culture of cell suspensions. Currently, *in vitro* culture systems involve base media and supplements with variations in protein source and growth factors, and the temperature in a CO_2_ incubator can vary from 31 to 37 °C, depending on the species.

A pioneering study conducted by our collared peccaries research group has established means and supplements for the development of spermatogenesis. In collared peccary, after 28-days *in vitro* culture, the StemPro-34 SFM seemed to be the most adequate medium for *in vitro* culture of prepubertal peccary testicular tissue. Moreover, the supplementation with glial-derived neurotrophic factor (GDNF, 10.0 ng/mL) seems to be essential for the maintenance of cell survival and proliferation (Silva et al., 2021b).

### Perspectives of preserving testicular tissues

Technologies for testicular tissue preservation are relatively recent compared to other ARTs. Despite this, numerous advances have been described around the world, to either develop cryopreservation protocols or to establish adequate culture systems. Birth of viable offspring from frozen testicular tissue and subsequently submitted to grafting protocols has already been described not only in mice (Shinohara et al., 2002), but also in domestic pigs (Kaneko et al., 2013) and primates such as the Rhesus monkey (Fayomi et al., 2019). These impressive results are encouraging examples for wildlife conservation.

## Final considerations

Over the past decades, biodiversity loss has accelerated at a global scale. To maintain and sustain biodiversity, ARTs have been developed and implemented by many researchers around the world. Cryopreservation of ovarian and testicular tissue, when properly performed, can be useful approaches for understanding the biology of germ cells in different species. It allows the evaluation of the mechanisms of contraceptive agents responsible for infertility, the correction of the causes of infertility by editing the genome of these cells, and the obtaining of viable sperm to be used in the embryo production.

In some species from the Caatinga biome, important progress has been made. Regarding the preservation of PAFs, it still has limitations when it comes to being a source of oocytes for ARTs in wild animals. On the other hand, the use of cryopreservation and *in vitro* culture of PAFs have been important tools for the knowledge of folliculogenesis in different species. Several protocols for cryopreservation and *in vitro* culture of PAFs have already been developed, and their results vary according to the conditions established for the preservation protocols, *in vitro* culture systems, media and supplements used, and, mainly, according to the investigated species.

Although testicular tissues from adult individuals have already been cryopreserved in different species, no protocol is effective enough to produce mature spermatozoa from cryopreserved testicular tissues. This fact highlights the need to continue studies to produce viable sperm and, consequently, live offspring. For this reason, steps to optimize SSV have been developed in collared peccaries and agoutis, wild mammals of the national fauna, with the aim of developing SSV as a safe bioprocess in the conservation of biodiversity.
